# Hemophagocytic Lymphohistiocytosis Complicating Erythroleukemia in a Child with Monosomy 7: A Case Report and Review of the Literature

**DOI:** 10.1155/2013/581073

**Published:** 2013-12-12

**Authors:** Samin Alavi, Maryam Ebadi, Alireza Jenabzadeh, M. T. Arzanian, Sh. Shamsian

**Affiliations:** ^1^Pediatric Congenital Hematologic Disorders Research Center, Shahid Beheshti University of Medical Sciences, Mofid Children's Hospital, Tehran 15468-15514, Iran; ^2^Tehran University of Medical Sciences, Tehran, Iran

## Abstract

Herein, the first case of childhood erythrophagocytosis following chemotherapy for erythroleukemia in a child with monosomy 7 is reported. A 5-year-old boy presented with anemia, thrombocytopenia, and hepatosplenomegaly in whom erythroleukemia was diagnosed. Prolonged pancytopenia accompanied by persistent fever and huge splenomegaly and hepatomegaly became evident after 2 courses of chemotherapy. On bone marrow aspiration, macrophages phagocytosing erythroid precursors were observed and the diagnosis of HLH was established; additionally, monosomy 7 was detected on bone marrow cytogenetic examination. In conclusion, monosomy 7 can lead to erythrophagocytosis associated with erythroid leukemia and should be considered among the chromosomal abnormalities contributing to the association.

## 1. Introduction

Hemophagocytic lymphohistiocytosis (HLH), a critical and severe disorder, is characterized by severe hyperinflammation resulting from proliferation of reactive lymphohistiocytes on the basis of various inherited or acquired immune deficiencies [1]. The entity is further classified into two subgroups: familial (primary) HLH and acquired (secondary) HLH [1]. Malignant neoplasm-associated HLH, a subtype of secondary HLH, is mainly accompanied by malignant lymphoma and, less frequently, by other hematological malignancies and carcinomas. HLH is known as a rare and adverse complication of childhood malignancies including acute myeloid leukemia (AML) [2]. There are only four reports available in the literature on the association of AML (M6) and HLH, all of which are described in adult patients [1, 3] (Table 1). A variety of chromosomal abnormalities are observed in these associations; however, monosomy 7 has not been previously reported in these cases. Herein, the first case of childhood HLH complicating erythroleukemia in a 5-year-old boy with monosomy 7 is reported.

## 2. Case Report

**Figure 1:**
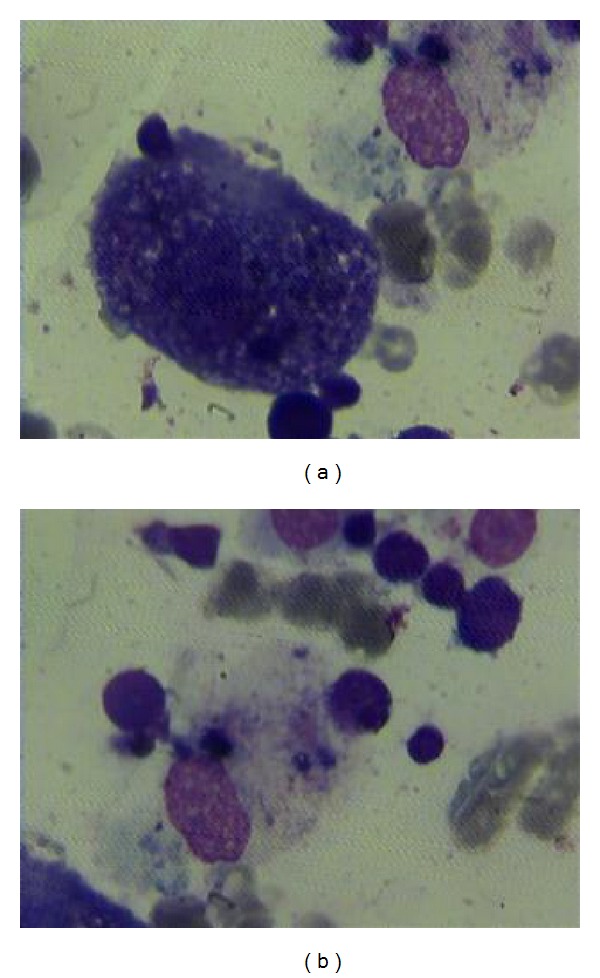
Bone marrow smears demonstrating phagocytosis of erythroblasts by macrophages in the bone marrow.

A 5-year-old boy was admitted to pediatric oncology department with anemia, thrombocytopenia, and hepatosplenomegaly. Bone marrow aspiration displayed erythroid hyperplasia with erythroid precursors forming more than 50% of the cell population. Megakaryocytes were decreased in number. On repeat bone marrow aspiration performed two weeks later, more than 50% of nucleated cells were erythroblasts accompanied by myeloblast population more than 20%, compatible with AML6a. Chemotherapy with DAT protocol (daunorubicin, cytarabine, and thioguanine) was initiated. Following 2 courses of DAT therapy, prolonged pancytopenia lasting for more than 2 months with no evidence of hematologic recovery was manifested. Persistent fever and huge splenomegaly and hepatomegaly (3 cm below costal margin) were new findings on physical examination. Bone marrow aspiration was performed which proved to be severely hypocellular. A noticeable finding at this point was macrophages phagocytosing other blood cells mainly erythroid precursors; however, no evidence of increased myeloblasts was detected (Figure 1). Flowcytometric study was not conclusive with only CD117 being positive. Bone marrow cytogenetic examination was positive for monosomy 7. According to the presence of prolonged fever, persistent huge splenomegaly and pancytopenia, and evidence of erythrophagocytosis in bone marrow aspiration, diagnosis of HLH was established. Serum ferritin which was increased to 3849 *μ*g/L, mild hypertriglyceridemia with 185 *μ*g/L serum triglyceride level, and hypofibrinogenemia (15 *μ*g/L) were in accordance with the diagnosis. As indicated by the huge splenomegaly and refractory pancytopenia, laparotomy was performed through which splenectomy, a wedge liver biopsy, and sampling of para-aortic lymph nodes were done. Pathology studies of all specimens demonstrated increased numbers of macrophages and brisk erythrophagocytosis. He was determined to receive therapy according to HLH-2004 protocol and was doing well. However, shortly after commencing HLH directed therapy, an episode of febrile neutropenia was developed and the patient succumbed to sepsis.

## 3. Discussion

Herein, the authors report the first case of childhood erythrophagocytosis following chemotherapy for erythroleukemia in a 5-year-old boy who was also discovered to bear monosomy 7 on cytogenetic studies.

Specific Karyotypic abnormalities have been described in several hematologic malignancies. Clonal cytogenetic abnormalities, including monosomy 7, are detected in two-thirds of patients with AML [4]. Shitara et al. reported erythroleukemia in a child with monosomy 7 [5]. Schroeder et al. described a case with glycogen storage disease type Ib, who developed acute myeloid leukemia with a classical monosomy 7 years after continuous treatment with granulocyte colony-stimulating factor [6]. Conversely to our case, none of the previous cases of erythroleukemia and monosomy 7 developed HLH [5]. Cytogenetic studies performed on patients with AML (M6) associated with HLH are suggestive of the presence of specific chromosomal abnormalities (Table 1). In the present study bone marrow cytogenetic examination revealed monosomy 7.

Hemophagocytic lymphohistiocytosis (HLH) is an extended entity enclosing a variety of macrophage-related disorders characterized by fever, pancytopenia, hepatosplenomegaly, and finally hemophagocytosis in bone marrow, liver, or lymph nodes [2]. Hemophagocytic syndromes associated with malignancy occur in two forms. First, hemophagocytic syndromes (MAHS) can present initially, masking various hematolymphoid malignancies, and second, they may complicate the initial course. Interestingly, different types of malignancies are categorized under each group; specifically, T-cell leukemias and lymphomas are often masked by a hyperinflammatory MAHS state, whereas B-cell leukemias and germ cell tumors are often complicated by MAHS. The patient presented here developed obvious erythrophagocytosis following chemotherapy for AML and also multiple doses of G-CSF he received during prolonged period of pancytopenia. In some cases of MAHS, bone marrow aspirate reveals blasts or dysplastic neutrophils phagocytosing other cells and in these cases specific chromosomal abnormalities are observed on cytologic studies [1]. In our patient macrophages were phagocytosing other blood cells mainly erythroid precursors. As described by Tsuji et al. acute erythroleukemia and concomitant HLH emerge almost simultaneously [7]. However, in our case erythroleukemia occurred prior to HLH, which could be interpreted in two ways; HLH occurring as a complication of chemotherapy and/or treatment with G-CSF or presence of similar symptoms between the two entities could lead to delayed diagnosis of HLH.

The management of MAHS remains controversial. High doses of corticosteroids are recommended; however, the exact mechanism of their beneficial effect is yet to be identified [8]. HLH-2004-like approach is most predominantly used, nonetheless, less toxic strategies targeted at specific inflammatory defects have emerged. The described patient received therapy according to HLH-2004 protocol.

In conclusion, HLH complicating AML remains a rare and life threatening entity and requires special attention. Our case brings up the argument that monosomy 7 in setting of erythroid leukemia could be complicated by erythrophagocytosis and should be considered among the chromosomal abnormalities contributing to the association.

## Figures and Tables

**Table 1:** Cases with AML associated with HLH.

Author	Patient	Country	Diagnosis	Initial presentation	Cytogenetics
Wong et al. (1991) [9]	Male, 64 years	Hong kong	AML (M6)	Not documented	Not documented
Kumar et al. (2000) [4]	Female, 8 months	USA	AML (M0)	HLH	Translocation (4; 7), deletion (12), Trisomy (19), deletion (5)
Tadmor et al. (2006) [10]	Male, 2 years	Israel	AML (M4)	HLH	Monosomy 17, deletion (5)
Lackner et al. (2008) [2]	Female, 16 years	Austria	AML (M2)	AML	Not documented
Lackner et al. (2008) [2]	Female, 9 years	Austria	AML (M2)	AML	Not documented
Kitagawa et al. (2009) [11]	Male, 53 years	Japan	AML (M6b)	Simultaneous manifestation	Trisomy 1, 11, 13, 14, monosomy 3, 4, 9, 10, 12, 15, 16, 17, 18, 19, 22 Deletion 5, 20
Tsuji et al. (2010) [7]	Male, 60 years	Japan	AML (M6)	Simultaneous manifestation	Trisomy 1, 3, 4, 6, 8, 21, add (9), (15), (16), (19), (20)
Wang et al. (2010) [3]	Female, 59 years	Japan	AML (M4)	HLH	Trisomy 3, 8; add (2)
Yamazaki et al. (2011) [1]	Male, 74 years	Japan	AML (M6)	AML	Monosomy 5, Trisomy 8; add (3), (21), del (4), (7)
Alavi (this case) (2013)	Male, 5 years	Iran	AML (M6a)	AML	Monosomy 7

## Abbreviations

AML: Acute myeloid leukemia
HLH: Hemophagocytic lymphohistiocytosis.
